# Effects of land use types on soil erodibility in a small karst watershed in western Hubei

**DOI:** 10.7717/peerj.14423

**Published:** 2022-12-01

**Authors:** Ting Luo, Wenjing Liu, Dong Xia, Lu Xia, Ting Guo, Yueyang Ma, Wennian Xu, Yue Hu

**Affiliations:** 1 China Three Gorges University, Hubei Provincial Engineering Research Center of Slope Habitat Construction Technology Using Cement-based Materials, Yichang, Hubei, China; 2 Ministry of Natural Resources, Key Laboratory of Urban Land Resources Monitoring and Simulation, Guangdong, Shenzhen, China; 3 China Three Gorges University, College of Art, Yichang, Hubei, China

**Keywords:** Karst, Small watershed, Land use, Soil erodibility, *K*-factor

## Abstract

**Background:**

Soil erosion is a severe problem in the karst watershed, and analysis of soil erosion at the watershed scale is urgently needed.

**Methods:**

This study tried to estimate the soil erodibility factor (*K*-factor) using the Erosion Productivity Impact Calculator (EPIC) nomograph and evaluate the spatial distribution of the predicted *K*-factor in a karst watershed. Soil properties and *K*-factors of five land use types (NF: natural mixed forest, CF: cypress forest, EF: economic forest, ST: stone dike terrace, VF: vegetable land) in the Xialaoxi small watershed were compared and key factors affecting erodibility were analyzed.

**Results:**

Results showed that (1) The erodibility *K*-factor was unevenly distributed within different site types and strongly influenced by anthropogenic activities. The soil *K*-factors of sample sites subjected to frequent human disturbance (ST, VF) were high, ranging from 0.0480-0.0520 t hm^2^ h/(MJ mm hm^2^), while the soil *K*-factors of natural site types (NF, CF, and EF) were low, ranging from 0.0436-0.0448 t hm^2^ h/(MJ mm hm^2^). (2) The soil texture in the Xialaoxi watershed was mostly loamy, and that of the agricultural areas frequently disturbed by agricultural practices (ST, VF) was silty loam. (3) Soil carbon fractions were affected by land use types. Soil organic carbon storage of NF and CF had strong spatial heterogeneity. The soil organic carbon (SOC) and labile organic carbon (LOC) of the two were significantly higher than those of the disturbed EF and cultivated land soil. (4) There was a synergistic effect between the soil properties and the *K*-factor. *K* was significantly negatively related to sand fractions (2-0.05 mm) and non-capillary porosity, while positively related to silt content (0.05–0.002 mm). Overall, changes in bulk density (BD), total porosity (TP), non-capillary porosity (NCP), texture, and organic matter content caused by natural restoration or anthropogenic disturbance were the main reasons for soil erodibility. Natural care (sealing) and construction of stone dike planting practices were effective ways to reduce soil erosion in small karst watershed areas of western Hubei.

## Introduction

Soil erosion is the most common process that alters the landscape and is a major contributor to land degradation (Belayneh et al., 2021). Sediment is conveyed with surface runoff and finally sinks into surface water and settles in streambeds when soil separation occurs. These erosion processes impair soil fertility and the yield of arable land along watersheds, endangering the stability and service functions of the ecosystem. Small watersheds are the fundamental units of large-scale water confluence and sediment export, as well as the primary targets of soil and water conservation management (Zhang et al., 2021). The Chinese government has stressed stringent management of land use in medium and small watersheds along the Yangtze River in recent years, adopting a series of environmental protection policies, such as the River Chief System and Yangtze River Protection Forest Engineering. However, geological disasters such as excessive rainfall, floods, and landslides occur in the middle and lower sections of the Yangtze River due to the extreme climate (Gao, Yang & Yang, 2013). In view of these severe threats, rational utilization of land resources is critical for preventing soil erosion in small watersheds.

Soil erodibility describes the ease with which soil can be eroded and indicates the soil’s susceptibility to denudation and transport caused by external erosion forces. The *K*-factor is an essential parameter in many soil erosion forecasting models and is primarily governed by inherent soil parameters such as particle size composition, moisture, bulk density, pore structure, and organic matter concentration (Radziuk & Świtoniak, 2021). The most widely accepted models for calculating soil erodibility *K*-factor are the USLE/RUSLE (Martínez-Murillo, Remond & Ruiz-Sinoga, 2020), EPIC (Wang, Zheng & Guan, 2016), and Shirazi’s equation (Lin et al., 2017). The USLE involves soil structure and infiltration level parameters, which are difficult to standardize, and there is no manual to refer to in China, whereas the Shirazi formula only considers the geometric mean particle size of the soil, increasing the uncertainty of the calculated *K*-factor (Beretta & Carrasco-Letelier, 2017). Considering the availability of data in the study area and the scientific nature of the calculation results, the erosion-productivity evaluation model (EPIC) proposed by Williams, Renard & Dyke (1983) was chosen for this study. It is more widely used and gives stable *K*-factor values (Yu et al., 2018; Deng et al., 2019).

Watersheds are the basic units of confluence processes and sediment transportation of large-scale water systems. In terms of basin-scale analysis of soil erosion, highlighting the impact of soil properties and external influencing factors on soil erodibility has attracted widespread attention in the southwest karst area of China. For instance, Liu et al. (2020) applied the EPIC model to analyze erodibility variations of agricultural abandonment in the typical karst catchment of Guizhou province and found that the *K*-factor in the TOC (total organic carbon)-rich soils (>2%) is mainly influenced by soil particle distribution, while that in the TOC-poor soils is mainly determined by SOM (soil organic matter). Feng et al. (2016) also found that tillage disturbances increase the average annual soil loss rate from 2006 to 2011 in a partially cultivated peak-cluster depression basin of northwestern Guangxi. Zeng et al. (2017) observed that the spatial evolution of the *K*-factor is affected by rocky desertification degrees and the lithological distribution belts of typical karst geomorphology. Ma et al. (2016) used the RUSLE model to assess the amount of soil erosion in typical mountainous karst basins sand believed that slope gradient is the main limiting factor affecting the level of soil erodibility. Previous studies have focused on the central area of the karst zone in Southwest China (Yunnan-Guizhou Karst Plateau), found that the erodibility *K*-factor is affected by surface vegetation, topography, climate, soil texture, land use, and so on (Li et al., 2020). In the karst hill region with weak karst development, however, the current state of soil erodibility in various land use types under the effect of human disturbance is not yet clear. Therefore, the study of it can further scientifically guide human activities and be conducive to the healthy development of the environment.

The Xialaoxi watershed is a typical subtropical karst hill basin and a first-level tributary at the boundary between the middle and upper reaches of the Yangtze River. There exists already the pressure of shortage of land resources, and unreasonable land use has exacerbated soil erosion. Soil erosion and sediment yield affect the aquatic environment of the Yangtze River. Therefore, to reveal the physical-chemical properties of soil and the sensitivity of soil erosion under the influence of different land uses in a small way, watershed typical land use types in the Xialaoxi watershed in the middle of the Yangtse River were selected in this study. The aim of this research was: (a) to compare soil properties among various land use types and evaluate the effect of land uses on soil erodibility based on the EPIC model; and (b) to identify key factors affecting soil erodibility at a small scale karst watershed. The study is expected to provide theoretical support for the development of soil erosion research and water and soil conservation work in karst watersheds in western Hubei, and promote the coordinated development of ecosystems in the Yangtze River Basin.

## Materials & Methods

### Study site and sample collection

The study area, the Xialaoxi watershed(30°46′24″−30°51′16″N, 111°14′23″−111°16′3″E), is located in western Hubei, central South China. The climate is subtropical monsoon, the annual average temperature is 16.9 °C and the annual mean precipitation is around 1164 mm. Affected by a strong monsoon climate in the middle reaches of the Yangtze River, 70%–80% of the rainfall occurs during the monsoon season (June to October), in which the single point rainstorm occurs frequently in July and August. Since the construction of the water conservancy project of the Gezhouba Dam in the 1980s, large areas of wasteland have been restored to artificial cypress forests. Based on field survey, forest land (artificial cypress forest) accounts for 85.30% of the watershed’s land use, while cultivated land, bare ground, and residential land account for 11.64%, 1.23%, and 1.82%, respectively (Liang et al., 2021). Stone terrace is the major water-soil retaining measure for slope cropland in the three gorges areas. The farmland is mainly planted with cash crops (such as *Zea mays*, *Brassica napus*, *Citrus reticulata*, *etc.*), which are scattered in the valley area along the mainstream. The upper and middle reaches of the basin are mainly covered by coniferous forests (*Platycladus orientalis*) and shrubs (*Rosaceae*, *Leguminosae*, *etc.*).

**Figure 1:**
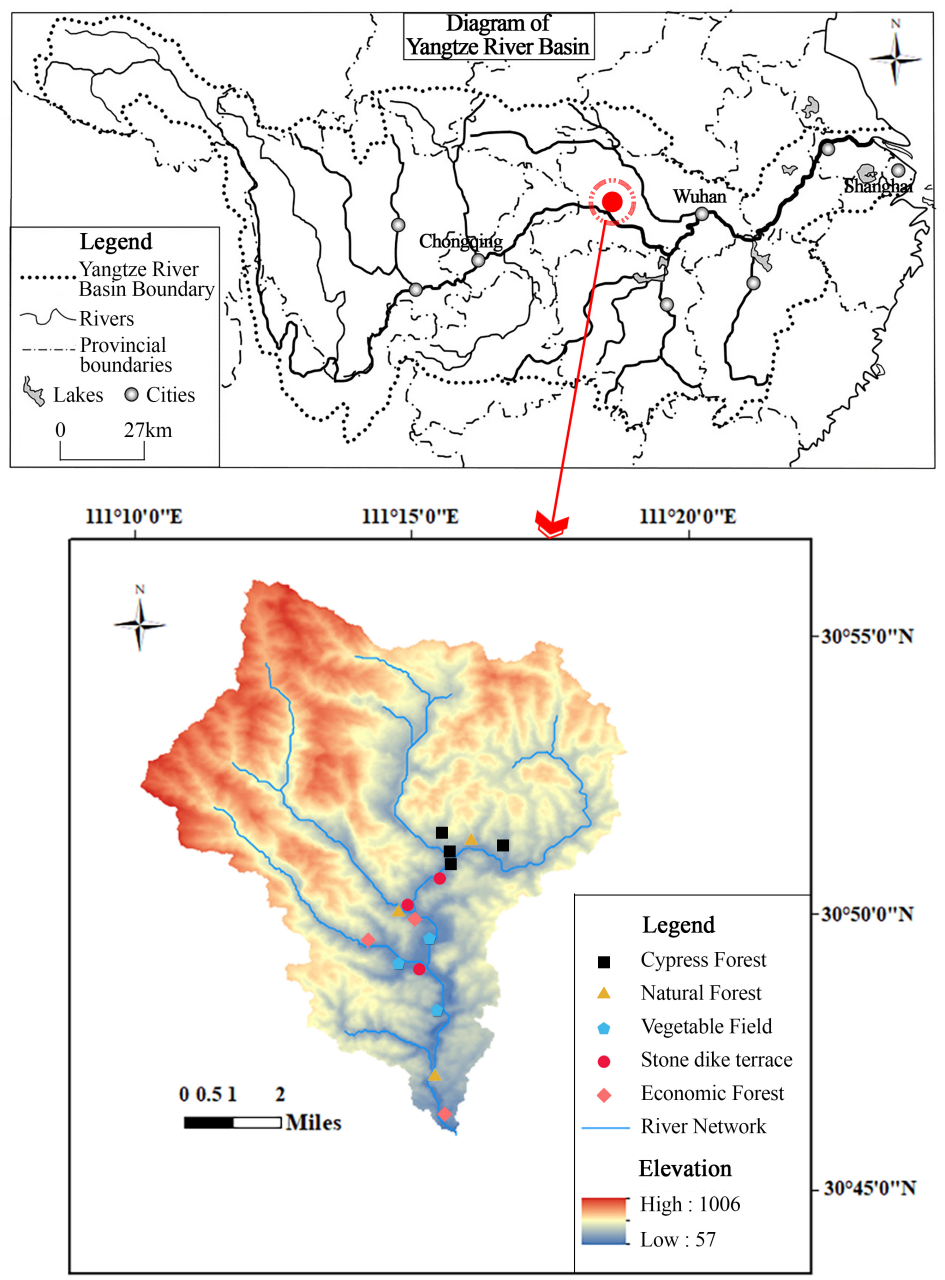
Location of the study area.

The field research was supported by Yichang Science and Technology Bureau (No. A20-3-010). From north to south, five typical land use types (natural mixed forest, cypress forest, economic forest, stone dike terrace and vegetable land, abbreviated as NF, CF, EF, ST, VF, respectively) were selected from major confluence areas as variable groups, three to four parallel plots of 20 × 20 m were chosen as the test sites for each. 16 sampling sites were selected for all five land use types in the whole watershed (Fig. 1). In each sample plot, undisturbed samples of 0–10 cm soil layer were taken for the determination of particle size distribution and soil organic carbon fractions. In addition, sampling of cylindrical metal cores (Φ50.46 × 50 mm, volume 100 cm^3^) were collected to measure the physical indexes of soil.

### Sample analysis

### Determination of soil physical properties

The samples were oven-dried at 105 °C for 24 h, and the ratio of oven-dry soil mass and volume of metal cylinders was calculated to measure bulk density (BD) (Amoakwah et al., 2017). Soil porosity and moisture capacity (SMC: Saturation moisture capacity, CMC: Capillary moisture capacity, NMC: Natural moisture content, TP: Total porosity, CP: Capillary porosity, NCP: Non-capillary porosity, PR: Pore ratio) were determined by the routing soaking method (Liu, 1996). Soil grain size was analyzed by the pipette method (Gregorich & Carter, 2007). Before measuring, samples were treated with 10% HCl and 9% H_2_O_2_ to remove carbonate and organic material.

### Analysis of soil carbon fractions and pools

Soil organic carbon (SOC) was determined through the dichromate oxidation method (Walkley & Black, 1934). Dissolved organic carbon (DOC) was determined by shaking 10 g of soil with 50 mL of 0.5 mol L^−1^ K_2_SO_4_ for 1 h (Jones & Willett, 2006). The extracts were filtered through a 0.45 µm membrane and analyzed by the wet digestion method. Labile organic carbon (LOC) was estimated using the method of 333 mM KMnO_4_ oxidation (Blair, Lefroy & Lisle, 1995), the variation of KMnO_4_concentration was used to estimate the amount of oxidized carbon, assuming that 1.0 mmol L^−1^ of MnO_4_ was consumed (Mn^7+^ →Mn^2+^) in the oxidation of 0.75 mmol L^−1^(9.0 mg) of carbon. Hardly oxidized carbon (HOC) was then determined as the difference between SOC and LOC. To obtain soil carbon storage of different land use types in the basin, organic C pool (Mg C ha^−1^) was determined by the following equation:

\begin{eqnarray*}\text{SOC stocks}({\mathrm{MgCha}}^{\text{- 1}})=[\mathrm{SOC}(\text{%})\times \mathrm{BD}({\mathrm{Mg~ m}}^{\text{- 3}})\times \text{Depth}(\mathrm{m})]\times 1{0}^{\text{4}}{\mathrm{m}}^{\text{2}}{\mathrm{ha}}^{\text{- 1}}\div 100(\mathrm{a}). \end{eqnarray*}

In the formula, the sampling depth in this study is the 0–10 cm layer.

### *K*-value estimation using EPIC model

In the Erosive Productivity Impact Calculator (EPIC) (Williams, Renard & Dyke, 1983) mode, soil erodibility *K*-factor is estimated based on SOC content and the percent of sand, silt, and clay as follows:

\begin{eqnarray*}\begin{array}{@{}c@{}} \displaystyle {K}_{EPIC}=\{ 0.2+0.3\exp \nolimits [-0.0256SAN(1-SIL/100)]\} \times ( \frac{SIL}{CLA+SIL} )^{0.3}\\ \displaystyle [1.0- \frac{0.25C}{C+\exp \nolimits (3.72-2.95C)} ]\times [1.0- \frac{0.7SN1}{SN1+\exp \nolimits (-5.51+22.9SN1)} ] \end{array} \end{eqnarray*}

where SAN is percent sand content, SIL is percent silt content, CLA is percent clay content, C is percent organic carbon content, and SN_1_ = 1−SAN/100. All results in the United States customary units were multiplied by 0.1317 to convert to the SI units for the *K*-factors (t hm^2^h (MJ mm hm^2^)^−1^).

### Statistical analyses

One-way ANOVA was used to evaluate the significance of differences in soil BD, porosity, organic C stocks, C fractions (DOC, LOC, HOC), soil erodibility *K* from different land use types. Multiple comparisons of the means were performed using the LSD test (*P* < 0.05). We also performed regression analyses of the correlations between *K*-factor and soil particle size to evaluate the statistical relationship between soil particle sizes and soil erodibility. To identify the influencing factors of soil erodibility, Pearson correlation analysis was used to analyze soil erodibility and soil properties. To screen the factors that have the greatest influence on soil erodibility *K*, a stepwise regression analysis was used to develop a multiple regression model for prediction. The dependent variable was soil erodibility *K*, and the independent variable was soil characteristic index. Data analyses were carried out with Excel and SPSS 24.0 software (SPSS Inc., Chicago, IL, USA).

## Results

### Soil pore characteristics

The characteristics of soil porosity under different land uses are shown in Table 1. The TP of the NF soil was the highest (57.00%), while the BD was the lowest (0.9 g/cm^3^), which indicated that the soil under natural forest had good permeability and strong capacity for water storage. Periodic tillage and scarification resulted in high TP and low BD in agricultural land (CF and ST). In comparison, the TP of the soil in EF was significantly lower than that of other land use types (*P* < 0.05), and the BD value was the highest (1.34 g/cm^3^), indicating that the soil structure was highly compact. The soil CP in VF (45.24%) and ST (44.96%) was significantly higher than that of forestry land, and the difference between the former two was not marked (*P* = 0.504). This was probably because that artificial cultivation practices such as ridge cultivation, loosening soil and irrigation, were conducive to the formation of non-capillary pores in the topsoil, and also helped crops to absorb water by capillary action. In addition, the NCP of soil in NF (14.64%) was significantly higher than that of other land use types (*P* < 0.05). Comparing the soil pore ratio under different land use types, it could be seen that the PR of NF was the highest (1.30), and the pore structure of EF was the worst (0.83). Different land use types affected the soil pore structure. On the whole, land utilization types significantly changed the total porosity (DF=4, *F* = 17.836, *P* = 0.001), and specifically the proportion of capillary pores in soil (DF =4, *F* = 6.032, *P* = 0.002) was affected.

**Table 1:** Distribution of soil porosity in different land use patterns in the small watershed. Values are means with a standard error. Different letters in the same column for each variable indicate the statistical significance of the effects of land use on physical properties of soil based on two-way analysis of variance.

Land uses	BD/g cm^−3^	TP/%	CP/%	NCP/%	PR
CF	1.05(0.08)bc	49.62(1.56)b	39.98(1.59)bc	9.63(1.32)b	0.99(0.06)b
EF	1.34(0.09)a	45.13(3.53)c	38.89(1.57)c	6.24(3.17)b	0.83(0.12)c
ST	1.12(0.06)b	53.80(2.67)ab	44.96(5.95)a	8.84(5.13)b	1.17(0.13)a
NF	0.99(0.09)c	57.00(2.56)a	41.76(2.42)bc	14.64A(2.88)a	1.30(0.15)a
VF	1.01(0.10)c	56.00 (2.62)a	45.24(3.47)a	10.76(0.97)ab	1.28(0.14)a

**Figure 2:**
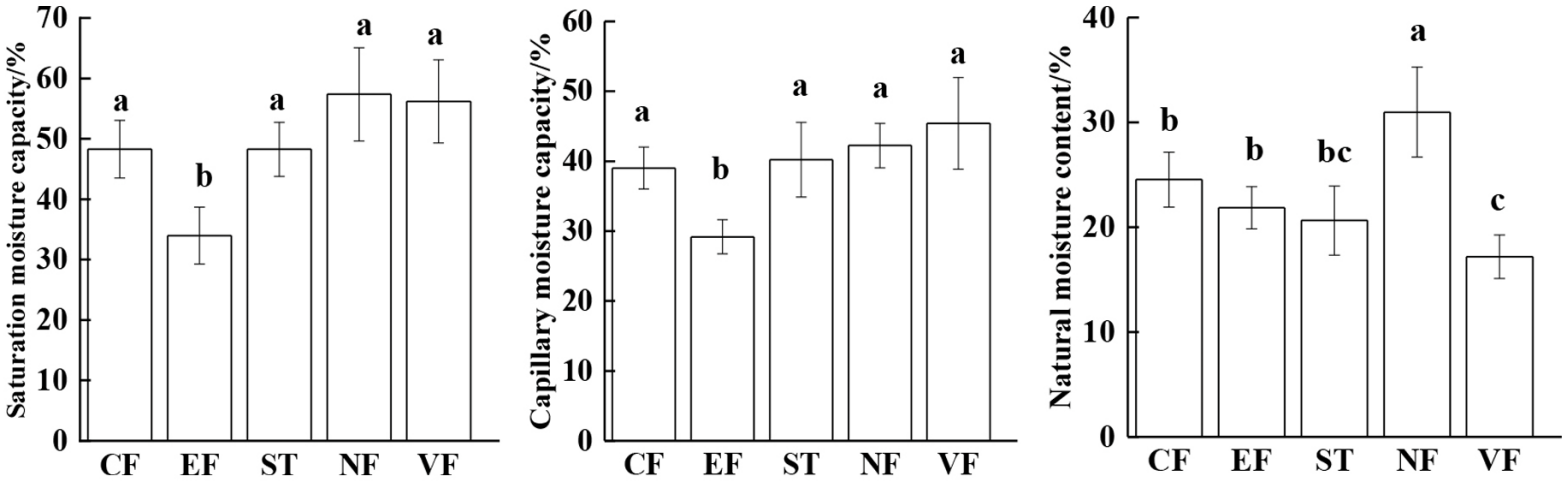
Soil water-holding capacity in different land uses. NF, natural mixed forest; CF, cypress forest; EF, economic forest; ST, stone dike terrace; VF, vegetable land. Different lowercase letters denote significant differences at *P* < 0.05.

The soil moisture contents among different land use types are shown in Fig. 2. In the 0–10 cm topsoil, SMC and CMC of EF soil were significantly lower than those of other land use types (*P* < 0.05), but there was no significant difference among the other four land use types (*P* > 0.05). The NMC in NF soil was the highest, 30.97%, while that of VF was the lowest, 17.18%. In general, the water-holding capacity of forest soil in the watershed was significantly higher than that of agricultural land.

### Soil organic carbon storage of different land uses

**Figure 3:**
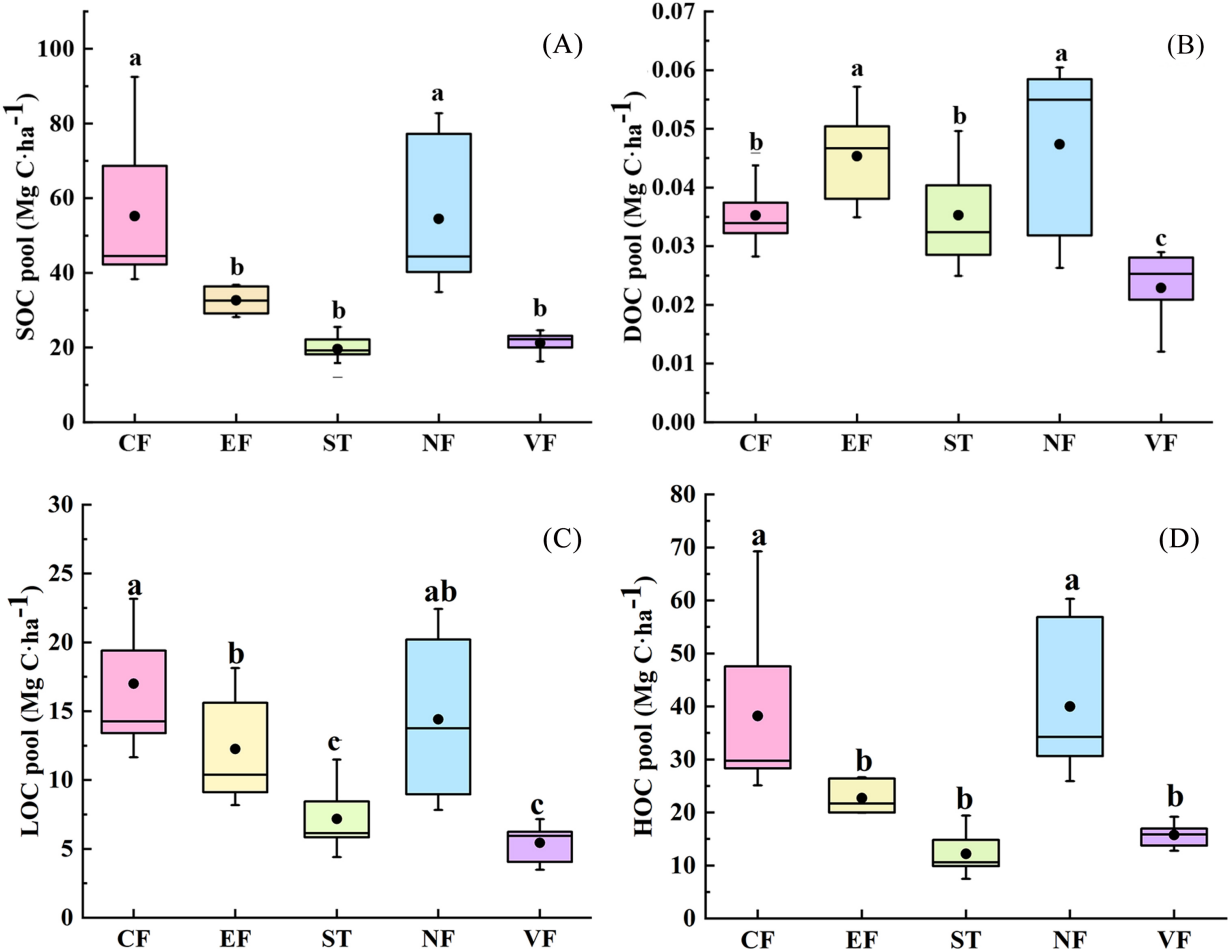
(A–D) Soil carbon fraction content of different land uses. NF, natural mixed forest; CF, cypress forest; EF, economic forest; ST, stone dike terrace; VF, vegetable land. Different letters denote significant differences at *P* < 0.05.

A significant difference was observed in the content of soil carbon fractions among land uses. Figure 3A shows that mean values of SOC storage from high to low were: CF >NF >EF >VF >ST. The average organic carbon storage of surface soil in litter-rich (CF and NF) forests was significantly higher than that of agricultural land severely affected by human disturbance (*P* < 0.05), with the former (54.80 Mg C ha^−1^) being nearly twice as high as the latter (23.51 Mg C ha^−1^). Strong variance was seen in the coefficients of variation of the SOC pools in NF and CF, which were 41.81% and 35.38%, respectively. The results indicated that the distribution of SOC stock in the forest lands was extremely uneven and had strong spatial heterogeneity within the watershed scale. In Fig. 3B, DOC storage in the topsoil of forest land was generally higher than that of agricultural land, obviously, land use types significantly affected the amount of DOC pools (DF = 4, *F* = 34.981, *P* < 0.05). As shown in Fig. 3C, compared with the EF, the carbon pool oxidation activity of perennial forest land with high canopy density was relatively stronger, but the active carbon pools of agricultural land in the basin were rather low (3.49–12.91 Mg C ha^−1^). Because of the rapid turnover of labile organic carbon in the process of the soil C cycle, the influence of external conditions on soil organic matter properties could be sensitively reflected. The difference of HOC concentration among different land uses was similar to that of SOC distribution (Fig. 3D), natural forest land has the largest inert carbon pool in all land use types. It could be seen that LOC and DOC had a strong response to the disturbance of agricultural cultivation and could sensitively evaluate the impact of external soil environment on organic carbon content and quality of soil.

### PSD and soil erodibility of different land uses

**Figure 4:**
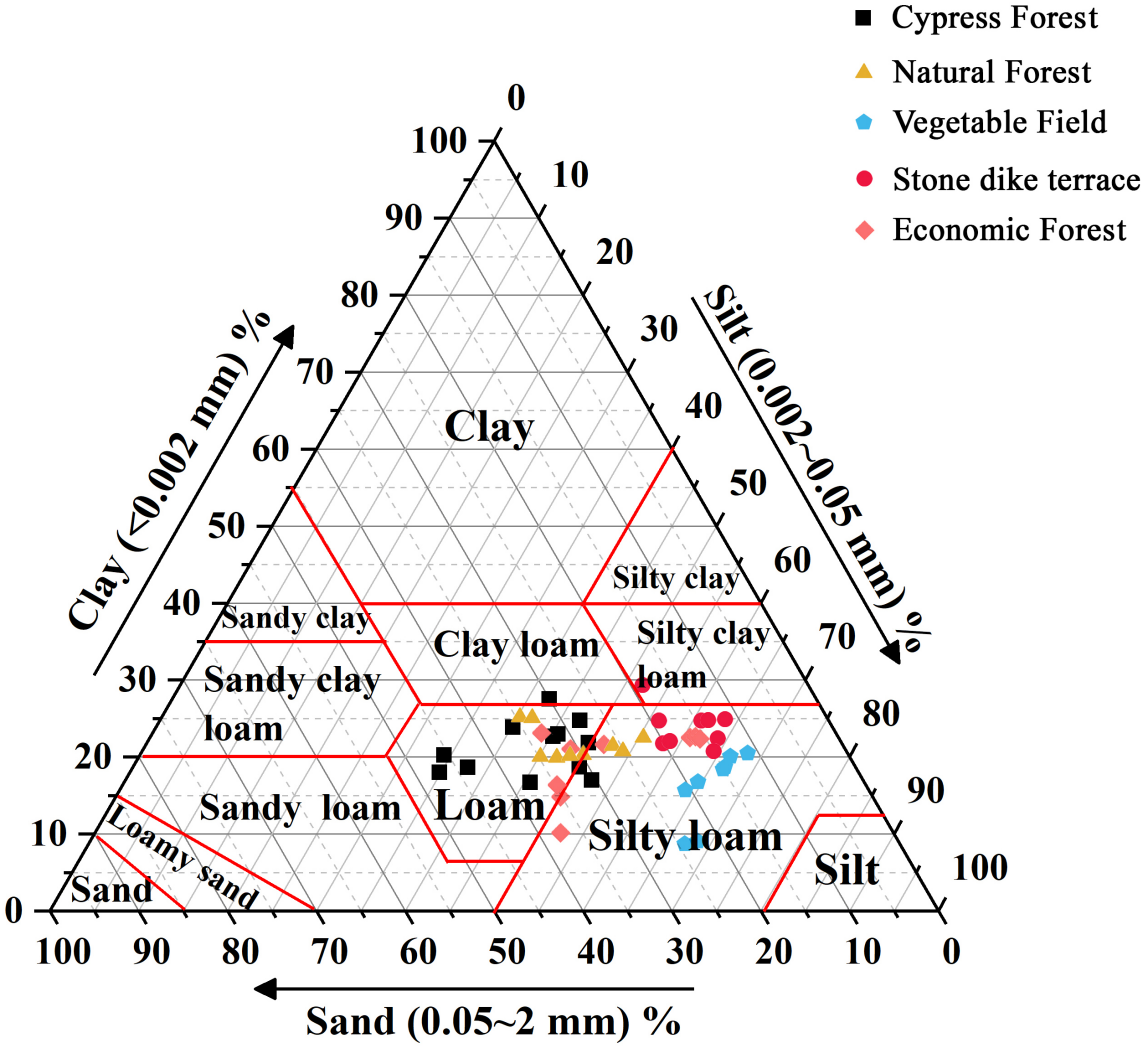
Soil texture under different land use patterns in the Xialaoxi watershed.

As can be seen from Table 2, there are considerable differences in PSD among the five land use types in this study. At the watershed scale, silt content (0.05−0.002 mm), accounting for 43.50%–67.68% of total particles, was dominant in deciding the soil particle size. Specifically, the silt content of VF (67.68%) was the highest and was significantly higher than that of the other land use types. The silt fraction proportions of the three types of woodland soil (NF, CF, and EF) were relatively small. For sand fractions, the content of sand (2−0.05 mm) in forestry-land soil was significantly greater than that in agricultural land uses. The sand fraction of forestland soil was primarily coarse sand (1−0.25 mm) and fine sand (0.25−0.05 mm). The proportion of gravel (2-1 mm) was the smallest, and the gravel content (2-1 mm) of CF soil was the highest among all land use types. This indicated that the soil texture of forestland was coarse, which was consistent with the limestone soil in karst area filled with rock debris, gravel, and weathered materials. Besides, the sand content of VF was significantly higher than that of ST. The clay content of ST accounted for 22.94%, the clay content of VF was the lowest, only accounting for 9.09%. According to the soil texture triangle map of the USDA (Condappa et al., 2008), the soil texture of small watershed was principally loam, and that of the agricultural land (ST and VF) frequently disturbed by farming was silty loam (Fig. 4).

**Table 2:** Particle size distribution and *K*_*EPIC*_ under different land use patterns.

Land use pattern	The percentage of soil particle size (ranges defined in mm)							Soil erodibility
	Gravel	Coarse sand		Fine sand	Sand	Silt	Clay	
	2.0-1.0	1.0-0.5	0.5-0.25	0.25-0.05	2.0-0.05	0.05-0.002	<0.002	*K*_EPIC_/Mg ha h/ (MJ mm ha)
CF	7.34a	7.06a	4.53a	16.83a	35.75a	43.50c	20.75ab	0.0436c
EF	3.18b	3.11b	3.18ab	23.13a	32.60a	49.44c	17.96b	0.0448c
ST	1.78b	1.85b	1.79b	10.37b	15.78c	61.28b	22.94a	0.0480b
NF	2.25b	3.14b	3.70ab	21.36a	30.45a	47.91c	21.64ab	0.0439c
VF	2.80b	4.03ab	5.31a	11.10b	23.23b	67.68a	9.09c	0.0520a

The soil erodibility *K* in the study area was estimated by using the EPIC empirical equation, and the distribution range of *K*-factor in the basin was 0.0436−0.0520, with the average being 0.0454. Soil erodibility values were the highest in VF, followed by ST, and the lowest appeared in the forest land, and no significant differences among the forest lands were detected. The results showed that under the same erosional forces, the surface particles in VF were more likely to be scoured and separated than those of ST, and were more likely to be lost under the effect of runoff. In contrast, the soil under CF and NF was less sensitive to erosion and had strong anti-erosion ability.

### Correlation between soil erodibility K-factor and soil properties

**Figure 5:**
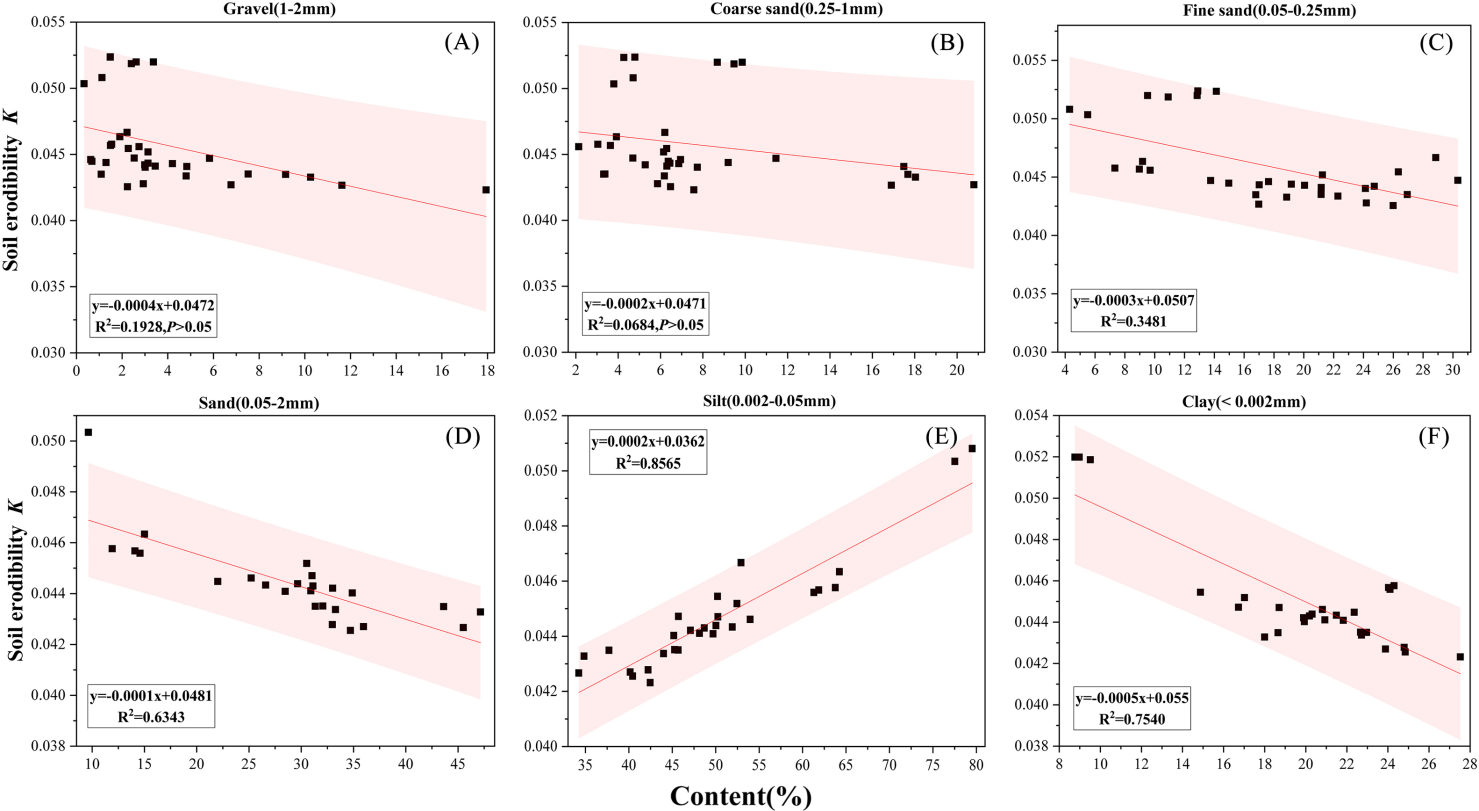
(A–F) Regression analysis between *K*_EPIC_ and coarse sand content.

Linear regression analyses were performed to determine the strength of correlation between *K*-factors and soil particle fractions. The statistical results showed that a highly positive correlation was found between soil erodibility and silt contents (Fig. 5E, *R*^2^ = 0.857, *P* < 0.01), and negative relationships were found between soil erodibility and sand content (Fig. 5D, *R*^2^ = 0.634, *P* < 0.01) and clay content (Fig. 5F, *R*^2^ = 0.754, *P* < 0.01). Soil with the high percentages of either sand or clay tended to be less vulnerable to water erosion. Similar result was observed for the fine sand content, which showed are relatively weak negative correlation with *K*-factors (Fig. 5C, *R*^2^ = 0.348, *P* = 0.03). However, no significant correlation was detected between *K* and the gravel content (Fig. 5A, *R*^2^ = 0.193, *P* = 0.08) and the coarse sand content (Fig. 5B, *R*^2^ = 0.068, *P* = 0.132).

**Figure 6:**
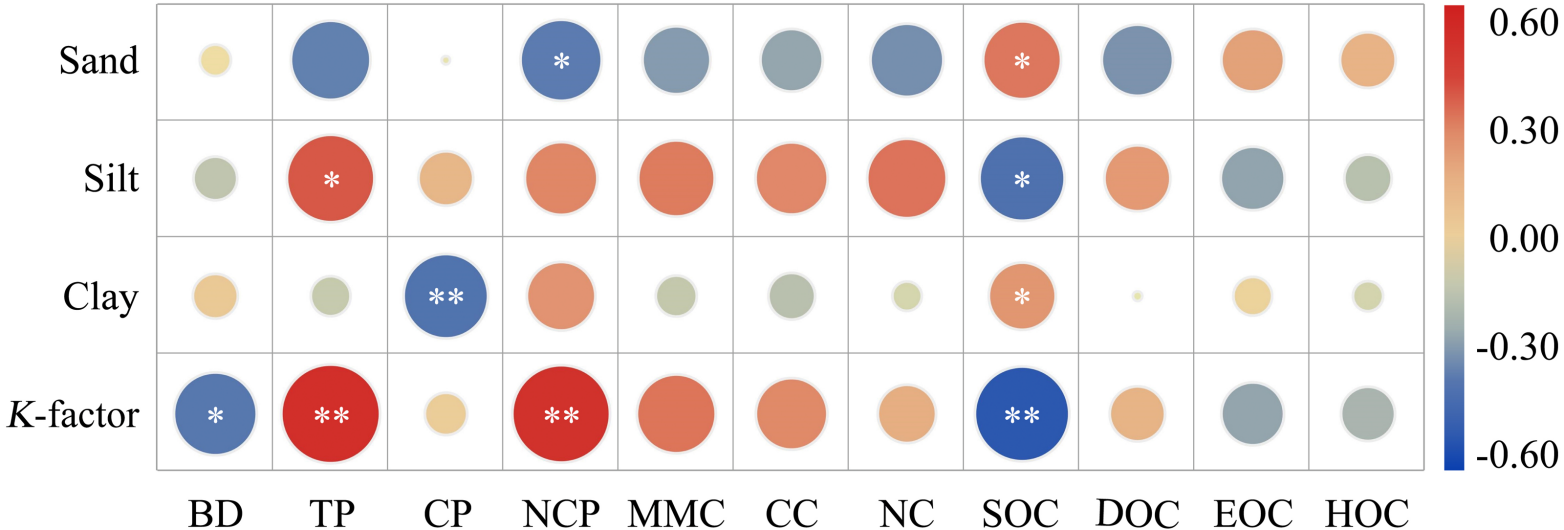
Correlation analysis matrix of soil erodibility and physical and chemical properties of soil. BD, buck density; TP, total porosity; CP, capillary porosity; NCP, Non-capillary porosity; MMC, max moisture capacity; CC, capillary capacity; NC, natural capacity; SOC, soil organic carbon; DOC, dissolved organic carbon; LOC, labile organic carbon; HOC, hardly oxidized carbon. * *P* < 0.05. ** *P* < 0.01, ns, not significant.

Land use type was the primary factor that directly changed the physical and chemical properties of soil, and further affected the sensitivity of surface soil to erosion. Therefore, to identify the influencing factors of soil erodibility, the correlation analysis between soil erodibility and soil properties was carried out (Fig. 6). As a result, *K*-factors were significantly positively correlated to TP and NCP, but negatively correlated to BD and SOC.

## Discussion

### Effects of land use on soil pore and water holding characteristics

Apart from characterizing soil water storage and permeability, BD and soil pore structure, as physical indicators for evaluating agricultural management practices, also reflect soil anti-erodibility (Mahalder et al., 2018). Compared with NF plots, soil BD of EF increased significantly. This is probably because artificial management of litter and weeds at the top layer at the site, as well as the overuse of chemical fertilizers, has resulted in low soil porosity. However, agricultural lands (VF and ST) are characterized by low BD and porous structures. During the process of periodic tillage, plowing increases soil porosity, particularly macropores, the soil becomes looser and BD decreases (Zhang, Zhao & Fu, 2017). In addition, the formation of CP in ST and VF soils is beneficial to crop water retention, probably affected by spraying water retaining agents during the dry season. NCP in the surface soil of NF was more than 10%, and PR reached 1.30 (Table 1), indicating that the soil pore structure is better. Restricted by shallow soil layer in the karst region of South China, the longitudinal roots of trees mainly extend horizontally on the surface, while the coarse roots penetrate horizontally inside the soil, which increases the non-capillary porosity and is more conducive to the soil water transmission (Yang et al., 2016). In general, different land use types could change soil porosity through tillage and surface plants. Natural vegetation is not affected by farming, and the interspersed roots system and covering of litter improve the non-capillary pores, which is conducive to precipitation infiltration, reducing ground runoff flow, and enhancing soil anti-scouring performance. Therefore, increasing the supervision and protection of natural vegetation could effectively prevent soil loss in the basin.

Soil saturated water holding capacity is composed of capillary moisture capacity and non-capillary moisture capacity. The soil BD and porosity influenced by different land use types have directly reflected the water holding capacity (Cai et al., 2020). According to the comparison in Fig. 1, compared with EF, the capillary moisture capacity of the soil of agricultural land was relatively higher, which might be resulted from the developed capillary porosity (Table 2). Capillary water of soil is restricted by capillary force and can be used by crop roots to absorb karst groundwater (Li et al., 2020). Due to the high degree of canopy density, the topsoil of NF and CF lands are inhibited from being exposed to direct light radiation, thus water evaporation loss in NF and CF forestland soil is reduced. Accordingly, the soil moisture content is significantly higher than that of agricultural land with poor cover conditions. In addition, the existence of abundant forest roots can conserve water resources.

### The variation characteristics of soil carbon pool under different land use types

The soil in the karst forest land is characterized by discontinuity, shallow soil layer, weak alkalinity, which lead to the accumulation and oxidation activity of SOC compared with other geomorphic areas (Bai & Zhou, 2020). SOC content is heterogeneous among different land types in the Xialaoxi watershed, the SOC stocks of arbor woodland (VF and CF), in particular, varies considerably with space. Field surveys have concluded that the litter quantity and root residues are the main sources of input to organic carbon pools of forest soil. However, the accumulation of soil organic carbon will be reduced after the original land and cover types are exploited artificially (Benbi et al., 2014). In this study, the average SOC of agricultural land (led by ST and VF) only accounted for 37.47% of the perennial woodland (NF). This is because when vegetation is destructed by conventional tillage practice, decomposition and erosion increase.

Besides SOC stocks, some research has demonstrated labile SOC fractions (*e.g.*, readily oxidation carbon and dissolved organic carbon), are more sensitive to erosion effects induced by soil management practices. Previous studies have shown that readily decomposable humic material, root exudates, and microbial polysaccharides are the main sources of soil active C pool (Almagro et al., 2013). In this study, NF and CF were the ecological non-commercial forest areas with abundant litter biomass and root density, supplying plenty of nutrients to the mineral soil layer through decomposition, leaching, and adsorption, which make the active C pools of NF significantly higher than that of EF and agricultural land. In addition, the EF is involved in the picking of fruits every year, resulting in a low level of passive C in the topsoil. The soil of ST and VF is frequently affected by the tillage erosion process, soil fine particles in eroded areas are reduced, macro-aggregates are broken, and active organic carbon in topsoil is more likely to be mineralized and lost. This is consistent with the study of Aichner et al. (2010), whose result is that SOC oxidative activity is reduced by farming in semi-arid basins. The bare surface environment may be conducive to the leaching of soluble carbon by rainwater, which is reflected in the relatively low concentration of DOC in the soil of agricultural land. Moreover, the DOC of ST is significantly higher than that of VF. Straw returning takes place in ST. After the straw is buried in the soil, the easily decomposed components (protein, soluble carbohydrate, *etc.*) decompose to produce a bit of monosaccharide, amino acids, amino sugars, and other small molecular substances, resulting in the rapid increase of soil DOC content. Furthermore, the priming effect caused by straw turnover also promotes the transformation of SOC into DOC.

### Effects of land uses on *K*-factor

Diverse land uses are the primary factors that change surface vegetation and soil quality. Due to the differences of vegetation characteristics and human disturbance during the process of soil management, the soil erodibility *K*-factor, which reflects the sensitivity of soils to erosion, is affected by land use (Deng et al., 2019). On the whole, the mean *K*-factor of woodlands was significantly lower than those of agricultural lands, indicating that soil subjected to years of tillage was more vulnerable to erosion. This is in agreement with the relevant studies of Dutal & Reis (2020) in the karst basin of the Mediterranean Highland. Two reasons can explain this: First, plant factors. Abundant surface land cover could guide run off infiltration. Because of the vertical structure of plant communities, the rainfall redistribution of NF and CF forests could be adjusted by heterogeneous canopy, understory, and litter layer, to reduce rainfall erosivity of the topsoil (Lacombe et al., 2018). The second reason is farming factors. The tillage disturbance caused the disintegration of soil macro-aggregates, exposing the internal organic carbon to the decomposition of microorganisms, thereby accelerating the mineralization of SOM and making the soil more susceptible to erosion (Ebabu et al., 2019). The *K*-factor of ST was significantly lower than that of VF (Table 2), indicating that stone terraces, as a typical soil and water conservation measure for slope cropland in the three gorges areas (Shen et al., 2010), divide the complete long slope into several transverse slopes. This can reduce the slope and fluctuation of local micro landforms, consequently weakening natural sediment yield and runoff erosivity (Xu et al., 2018).

The disintegration of macro-aggregates and the fine particle loss in soil are indicative of the deterioration of soil structure, this can be attributed to irrational human activities (Xu, Li & Peng, 2013). Thus, identification of soil PSD may provide an indicator for sustainable land utilization (Quijano, Kuhn & Navas, 2019). The study found that the soil texture of different land use types in the basin was mainly loam, while the cultivated soil (ST, VF) was silty loam with relatively fine texture, indicating that the land use types significantly affect the soil texture. Some academic studies have also found that the soil particle composition is also correlated to the vegetation feature (Xia et al., 2020), micro-topography (Rodríguez-Lado & Lado, 2016), anthropogenic interference (Sun, Liu & Xue, 2016) caused by land use alteration. In the study area, the vegetation cover affects erosion by intercepting rainfall and reducing the capacity for wind and water to be transported to the sediment, thus retaining fine particles (Meng et al., 2018). Moreover, the root exudations and abundant SOM from understory humus promote the formation of clay in NF and CF. In contrast, the bare land in VF has been frequently subjected to tillage disturbance, and clay fractions decrease sharply after runoff erosion. Man-made stonewalled terracing measures can transform the topography of sloping farmland into the flat terrace, which is conducive to more slope surface seepage (Xu et al., 2018). As a result, richer clayey structure has been developed compared with vegetable soil. Overall, land use types in the watershed are mainly responsible for differences in vegetation cover, micro-terrain, and man-made disturbances, resulting in differences of soil PSD. In future research, the differences of soil properties under the joint action of various influencing factors should be comprehensively considered to support the research on spatial variability in the watershed.

### The response of soil properties to erodibility

Soil erodibility, as an important parameter reflecting soil resistance to erosion, largely depends on its properties (Brito et al., 2019). SOC and particle fractions, as the parameters in EPIC equation, directly constrain the *K*-factor. More specifically, many other soil properties can indirectly affect the *K*-factor by affecting SOM accumulation and soil PSD (Wang et al., 2018). Our research suggested that SOC was negatively correlated with *K* (*r* =  − 0.513, *P* < 0.01). Humus produced by the decomposition of organic residues is liable to combine with metal cations (such as Ca^2+^, Fe^3+^) in soil, promoting the adhesion of soil fine particles, improving the soil aggregate stability and the soil anti-erosion (Man, Gh & Qian, 2019). However, Bonilla and Johnson (Bonilla & Johnson, 2012) have found that there is no relationship between organic matter and soil erodibility in the Central Valley region of central Chile. Previous works have believed that SOC alone could not accurately reflect erodibility, because there are mutual constraints between SOC and other factors, such as soil texture and permeability (Jin et al., 2009).

Among the physical properties of soil, percentage of mineral particles (sand, silt, clay) appears to have the highest correlation to soil erodibility. It was observed that the sand ( *r* =  − 0.798) and clay contents (*r* =  − 0.867) were negatively correlated with *K*-factors under different land use types (*P* < 0.01). Silt, contrary to sand and clay, affected the soil erodibility (*r* = 0.925) positively. Bonilla & Johnson (2012) found erodibility decreases with the increase of sand content, and the opposite is true for silt. Silt appears to be one of the major factors of vulnerability of the soil to water erosion. In our study, the highest soil erodibility had been observed in soils containing predominantly silt, which is consistent with the findings of Vaezi, Hasanzadeh & Cerdà (2016). In karst regions, the coarser soil texture means that the macropores of the soil are more developed, which is conducive to the infiltration of surface water into deep layers and the weakening of erosivity of overland flow. Clay minerals persistent bonding agents widely exist in soil. It is commonly recognized that clay particles are easy to combine with SOM to form water-stable aggregates, thus reducing the detachment capacity of soil particles and improving the erosion resistance of soil (Zhu et al., 2020). Nevertheless, some held that augment of fine particles blocks the soil hydrophobic pores (Zhou et al., 2015). A rapid decrease in infiltration rate has been observed during sequence rainfalls due to high antecedent moisture content, thus increases the rate of sediment yield. To some extent, to reflect soil erodibility only from a certain characteristic grain size of soil is not adequate because in the composition of soil particles, an increase in a specific particle size reduces the fraction of another particle size. This reveals there is a synergistic effect between the soil properties and the *K*-factor (Romero, Stroosnijder & Baigorria, 2007). Moreover, the relevant studies have shown that *K*-factors are more associated with soil properties when soil properties are not considered in isolation (Ayoubi et al., 2018). Consequently, it is more reliable to analyze erodibility based on soil texture than merely considering a single particle size.

**Table 3:** Stepwise regression analysis of relationship between erodibility and soil properties.

Factors	Unstandardized coefficient	Standard error	Standardized coefficient	T-value	*P*-value
Constant	0.145	0.053	–	2.763	0.011
Silt	0.268	0.068	1.210	3.940	0.001
NCP	−0.002	0.001	−0.466	−3.606	0.001
Sand	−0.151	0.064	−0.749	−2.368	0.026

Soil erodibility has been proven to be the result of complex reactions between many soil properties that contribute significantly to soil loss (Zhu et al., 2019). To ensure the independence of *K*-factor variables, the minimum set of soil properties for evaluating erodibility has been established by the stepwise multiple regressions. As shown in Table 3, *K*-factor is related to sand, silt, and NCP with a R^2^ of 64.9% as shown in the following equation:

\begin{eqnarray*}K=-0.151\times San+0.268\times Sil-0.002\times NCP+0.145{R}^{2}=0.649,F=15.430,p\lt 0.01. \end{eqnarray*}

Where, *K* is soil erodibility (t hm^2^ h (MJ mm hm^2^)^−1^), sand and silt areas are the percentage in soil particles. This model explains about 64.9% of soil *K* variance in the karst watershed, about 43.8%, 13.3%, and 7.80% of *K* variance in silt, NCP, and clay, respectively. As a result, in optimization and sustainable management, appropriately reducing the crushing of coarse particles (2−0.05 mm) and maintaining soil macropores can effectively decrease soil’s vulnerability to erosion in the karst watershed.

## Conclusions

Soil texture of the Xialaoxi watershed was mostly loamy, and that of the agricultural areas frequently disturbed by agricultural practices (ST and VF) was silty loam. The *K* ranged from 0.0436 to 0.0520t hm^2^ h/(MJ mm hm^2^), with a mean of 0.0454. The *K*-factor of watershed was significantly affected by land use type, and had obvious spatial heterogeneity. Overall, VF had the highest etch ability *K*-factor, followed by ST, and the lowest *K*-factor was detected for CF, EF, and NF. After NF was reclaimed into cultivated land (VF and ST) and economic forest land, soil pore structure and water holding capacity worsened. Therefore, resisting the reclamation of native forest areas and maintaining the tillage methods of stone dike terraces have positive benefits for the soil maintenance of the basin. Agricultural farming activities have reduced the soil erosion resistance, so it is necessary to properly take some soil erosion conservation measures on cultivated land, such as building hedges to reduce soil erosion.

Soil carbon fractions were affected by land use types. SOC storage of NF and CF had strong spatial heterogeneity in the karst basin. The SOC and LOC of the two were significantly higher than those of the disturbed EF and cultivated land. In addition, stepwise regression analysis showed that soil texture and non-capillary porosity in this region were the determinative factors for the *K*-factor. More specifically, *K* was significantly negatively related to sand fractions (2−0.05 mm) and non-capillary porosity, and positively related to silt content (0.05−0.002 mm). Furthermore, aside from the intrinsic properties (*e.g.*, texture, SOC, *etc.*) and temporary properties of soil (BD, moisture content, *etc.*), research on soil erosion in karst watershed should also look into more external factors (watershed hydrologic parameter, gradient, climate conditions, *etc.*), and this probably could be the focus of future research.

##  Supplemental Information

10.7717/peerj.14423/supp-1 Data S1 Raw data

## Additional Information and Declarations

### Field Study Permissions

The following information was supplied relating to field study approvals (*i.e.*, approving body and any reference numbers): Field experiments were approved by the Science and Technology Bureau of Yichang City, China, project number: A20-3-010.

### Data Availability

The following information was supplied regarding data availability: The raw measurements are available in the Supplementary File.
